# Rosiglitazone Inhibits TGF-β 1 Induced Activation of Human Tenon Fibroblasts via p38 Signal Pathway

**DOI:** 10.1371/journal.pone.0105796

**Published:** 2014-08-21

**Authors:** Yong-Heng Luo, Ping-Bo Ouyang, Jiao Tian, Xiao-Jian Guo, Xuan-Chu Duan

**Affiliations:** Department of Ophthalmology, Second Xiangya Hospital, Central South University, Changsha, Hunan Province, China; University of California, Merced, United States of America

## Abstract

**Purpose:**

Transdifferentiation of human Tenon fibroblasts to myofibroblasts and subsequent deposition of extracellular matrix is a key step in the scarring after glaucoma filtration surgery. The p38 signaling pathway plays an important role in cell proliferation and differentiation, and its upstream regulators and downstream molecules are widely distributed in the eye. We aimed to investigate the role of p38 in the activation of Tenon fibroblasts and that of the anti-fibrotic mechanism of rosiglitazone in the modulation of the p38 signaling pathway.

**Methods:**

Cultured Tenon fibroblasts were stimulated with transforming growth factor (TGF)-β1. Activation of p38 was examined by western blot analysis. Rosiglitazone and blocking of the p38 signaling pathway by SB203580 were used to antagonize stimulation by TGF-β1. Fibroblast motility was examined by wound closure assay; alpha-smooth muscle actin, connective tissue growth factor, and collagen type I were determined by qPCR and western blot. Expression and localization of alpha-smooth muscle actin were determined by immunofluorescence staining.

**Results:**

Phosphorylated p38 was upregulated in fibroblasts stimulated with TGF-β1, and this effect was substantially inhibited by rosiglitazone. Proliferation and migration of fibroblasts were suppressed by rosiglitazone and SB203580. Expression of alpha-smooth muscle actin, connective tissue growth factor, and collagen type I were decreased at the mRNA and protein levels by rosiglitazone and SB203580. However, the inhibitory effect of SB203580 on transcription and protein expression was weaker than that of rosiglitazone. Similar phenomena were found on immunofluorescence microscopy of alpha-smooth muscle actin.

**Conclusions:**

The p38 signaling pathway mediates the TGF-β1-induced transdifferentiation of human Tenon fibroblasts to myofibroblasts. Rosiglitazone can exert anti-fibrotic activity by interfering with the TGF-β/p38 signaling pathway and might be useful for modulating scar formation after glaucoma filtration surgery.

## Introduction

Postoperative scarring is the most common reason for failure of glaucoma filtration surgery. The transdifferentiation of human Tenon fibroblasts to myofibroblasts and subsequent deposition of extracellular matrix are key steps in the scarring of the filtration passage. The persistence of myofibroblasts, characterized by synthesis of α-smooth muscle actin (α-SMA), leads to hypertrophic scar formation and obstruction of the filtration passage. Transforming growth factor (TGF)-β is a key mediator of the transdifferentiation of fibroblasts to myofibroblasts [1], [2]. TGF-β is involved in a variety of biological actions, such as cell differentiation, osteogenesis, hematopoiesis, and inflammatory reaction [3]. Blocking of TGF-β may suppress the immune response and hinders wound healing. However, blocking of fibrosis-associated TGF-β signal transduction pathways [4], [5] may be a safe and effective way to regulate postoperative scarring.

TGF-β can activate mitogen-activated protein kinase (MAPK) in a cell-type–dependent manner [6], [7]. Moreover, MAPK pathways activated by TGF-β1 play a role in TGF-β–stimulated collagen type I (Col I) expression in certain cells [8], [9]. The p38 MAPK signaling pathway plays an important role in cell proliferation and differentiation, and its upstream regulators and downstream molecules are widely distributed in the eye. Peroxisome proliferator–activated receptor-γ (PPAR-γ), which belongs to nuclear hormone receptor superfamily of ligand-activated transcription factors, has been found to regulate fibrosis in multiple organs [10], [11]. Our previous study demonstrated that rosiglitazone, a synthetic PPAR-γ agonist, could moderately antagonize TGF-β1–induced fibrosis by interfering TGF-β/Smad pathway in vitro [12]. However, the mechanism of p38 pathway involvement in fibrosis after filtration surgery has not been fully clarified.

The aim of this study is to investigate the role of p38 MAPK in the activation of Tenon fibroblasts and anti-fibrotic mechanism of rosiglitazone in the modulation of the TGF-β/p38 signaling pathway.

## Methods

### Reagents

We purchased anti–α-SMA, horseradish peroxidase–conjugated immunoglobulin (Ig) G secondary antibodies, fluorescein isothiocyanate (FITC)-labeled IgG secondary antibody, and goat anti-mouse IgG from Proteintech (Chicago, IL). Antibodies to connective tissue growth factor (CTGF) and Col I were obtained from Santa Cruz Biotechnology (Santa Cruz, CA). Antibodies to vimentin, cytokeratin and glyceraldehyde-3-phosphate dehydrogenase (GAPDH) was obtained from Sigma (St. Louis, MO). Phosphorylated and non-phosphorylated antibodies to p38 were purchased from Cell Signaling Technology (Beverly, MA).

The p38 inhibitor SB203580 was purchased from Merck (Germany). A stock solution of SB203580 was prepared in dimethyl sulfoxide (DMSO). SB203580 was diluted in Dulbecco modified Eagle medium (DMEM; Gibco, Rockville, MD) and was added to the cell culture 1 h before stimulation with TGF-β1. Recombinant TGF-β1 was obtained from Peprotech Inc. (Rocky Hill, NJ) and used at a concentration of 5 ng/mL. A stock solution of rosiglitazone (Cayman, Ann Arbor, MI) for cellular assays was prepared in DMSO and then diluted in the optimal medium to a concentration of 10 µmol/L. The final concentration of DMSO in medium was less than 0.1%, and DMEM with 0.1% DMSO was used as vehicle control.

### Cell culture and phenotyping

The biological study followed the tenets of the Declaration of Helsinki and was approved by the Medicine Human Ethics Committee of the Second Xiangya Hospital of the Central South University. Human Tenon tissues were obtained from 3 selected patients during glaucoma filtration surgery after signed informed consent was obtained. Primary fibroblasts were harvested from an expansion culture of the Tenon tissues and were propagated in DMEM (Gibco) supplemented with 10% fetal bovine serum (FBS; Gibco, Rockville, MD), 100 U/mL penicillin, and 100 µg/mL streptomycin. Fibroblasts were identified by the immunofluorescence staining of vimentin and cytokeratin. Cells were labeled with mouse monoclonal antibodies against vimentin (1∶200; primary antibody) or mouse monoclonal antibodies against cytokeratin (1∶100; primary antibody) and then FITC-labeled goat anti-mouse IgG (1∶100; secondary antibody). Propidium iodide was used as a red counterstain. Human Tenon fibroblasts from passages 3 to 5 were used for all experiments. The experiments were performed at least three times in cell strains from the 3 patients and obtained similar results.

### Cell Counting Kit-8 assay

The activity of living cells was determined using Cell Counting Kit-8 assay (CCK-8; Dojindo Molecular Technologies Inc., Gaithersburg, Md.) according to the manufacturer’s instructions. Fibroblasts were seeded on 96-well plates at a density of 5×10^3^ cells/well and were treated with rosiglitazone for 2 h or SB203580 for 1 h in serum-starved medium. Then, the cells were incubated in serum-free medium with 5 ng/mL TGF-β1, mixture of rosiglitazone and TGF-β1, mixture of SB203580 and TGF-β1, SB203580 for another 24 h. After that, 10 µL monosodium salt was added to each well and incubated at 37°C for a further 4 h. The colorimetric absorbance was recorded at 570 nm, by using a model 3550 microplate reader (Bio-Rad Laboratories, Richmond, CA).

### Motility assay

Human Tenon fibroblasts were seeded in six-well plates, and scratch wounds were induced as described previously [12]. After suspended particles were washed away, we pretreated the cells with rosiglitazone for 2 h or SB203580 for 1 h in serum-starved medium. Then, the cells were stimulated with 5 ng/mL TGF-β1 in the presence of rosiglitazone or SB203580 for another 24 h. The wounds were monitored using phase-contrast microscopy. Width of wound was measured at six different sites and averaged in each sample. The width in each group was expressed as a percentage of the original measurement before stimulation, and the original width was presented as 100%.

### Quantitative real-time polymerase chain reaction

Fibroblasts were seeded in cell-culture flasks and grown to subconfluence. The cells were treated with treated with rosiglitazone for 2 h or SB203580 for 1 h. Next, the cells were incubated with 5 ng/mL TGF-β1 in the presence of rosiglitazone or SB203580 for 12 h (for CTGF mRNA analysis) or 24 h (for α-SMA and Col I mRNA analysis). The total RNA was harvested using Trizol according to instructions of the manufacturer (Invitrogen, Carlsbad, CA). After RNA gel electrophoresis, the qualified RNA was subjected to reverse transcription using the PrimeScript RT reagent kit (Takara, Shiga, Japan). Polymerase chain reaction (PCR) conditions were initial denaturation at 95°C for 10 min, followed by 40 cycles at 95°C for 15 s, 55°C for 20 s, and 72°C for 20 s. Amplification of the housekeeping gene (GAPDH) mRNA, which served as the internal control, was carried out with forward (CATCCTGCGTCTGGACCTGG) and reverse (TCCACCACCCTGTTGCTGTA) primers. The forward and reverse primer sequences were AGCAGCCCAGCCAAGCACTG and GCCGGCCTTACAGAGCCCAG for α-SMA, CGGTGTACCGCAGCGGAGAG and CAGGGCTGGGCAGACGAACG for CTGF, and CGCATGAGCGGACGCTAACC and TTCCTCTTGGCCGTGCGTCA for Col I. The relative transcriptional levels were calculated as E = 2^−△△Ct^, where E is the gene expression value, and △Ct is the difference in critical threshold cycle (Ct) value between GAPDH and each gene.

### Western blot

Fibroblasts were treated with rosiglitazone for 2 h or SB203580 for 1 h. Next, the cells were incubated with 5 ng/mL TGF-β1 in the presence of rosiglitazone or SB203580 for 24 h (for CTGF analysis) or 48 h (for α-SMA and Col I analysis). Cell proteins were extracted using lysis buffer (20 mM Tris, 150 mM NaCl, 1 mM EDTA, 1% Triton X-100) supplemented with phosphatase inhibitors (1 mM sodium vanadate, 50 mM NaF) and protease inhibitors (0.1% phenylmethylsulfonyl fluoride; Complete protease inhibitor, Roche). After being boiled in Laemmli sample buffer, 10 µg of protein extracts were subjected to sodium dodecyl sulfate polyacrylamide gel electrophoresis (SDS-PAGE). Next, proteins were transferred to a polyvinylidene difluoride membrane by a Bio-Rad gel-blotting apparatus (Bio-Rad, Hercules, CA, USA). The blots were blocked with TBST (10 mM Tris HCl, 150 mM NaCl, 0.1% Tween-20) containing 5% non-fat dry milk, for 1 h at room temperature, and then incubated overnight with the desired primary antibodies at 4°C. After each incubation, membranes were washed thrice for 10 min each with TBST. Peroxidase was detected by chemiluminescence and visualized by exposure to X-ray films (Kodak, USA). Quantification of western blot products was performed by densitometry using the Quantity-One software (Bio-Rad), and presented as the ratio between the optical density of target protein band and the optical density of total p38 or GAPDH band (phosphorylated-p38/total p38 or target protein/GAPDH).

### Immunofluorescence staining of α-SMA

Fibroblasts were grown on coverslips at 37°C for 1–2 days before the study. After serum deprivation for 12 h, cells were preincubated with rosiglitazone for 2 h or SB203580 for 1 h in serum-starved medium. 0.1% DMSO was used as vehicle for 2 h. Subsequently, the cells were incubated with TGF-β1 in the presence of rosiglitazone or SB203580 for another 48 h. Fibroblasts were grown on coverslips at 37°C for 1–2 days before the study. After serum deprivation for 12 h, cells were preincubated with rosiglitazone for 2 h or SB203580 for 1 h in serum-starved medium. 0.1% DMSO was used as vehicle for 2 h. Subsequently, the cells were incubated with TGF-β1 in the presence of rosiglitazone or SB203580 for another 48 h. After being rinsed in phosphate-buffered saline, the cells were fixed in 4% paraformaldehyde, permeabilized by triton X-100, and blocked in phosphate-buffered saline buffer supplemented with 5% bovine serum albumin. Cells were labeled with mouse polyclonal antibodies against α-SMA (1∶200; primary antibody) and FITC-labeled goat anti-mouse IgG (1∶100; secondary antibody). Cells were viewed under a fluorescence microscope (Axio Vert 200, Zeiss, Germany).

### Statistical analysis

All data are presented as means ± SD. Statistical analyses were performed with SPSS 16.0 (Chicago, IL, USA). Comparisons between results from multiple treatment groups were performed using one-way analysis of variance, and Tukey post hoc test was performed to show the individual differences. Statistical significance was defined as *P*<0.05.

## Results

### Human Tenon fibroblasts phenotyping

**Figure 1:**
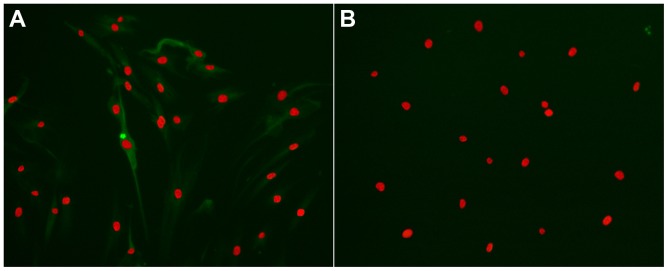
Immunofluorescence identification of human Tenon fibroblasts. Cells were identified by immunofluorescence with vimentin and cytokeratin. Propidium iodide was used as a nuclear counter stain. (**A**) Immunofluorescence double-labelling of vimentin (green) and the nuclear (red). (**B**) Cells did not express cytokeratin.

The fibroblasts are characterized by expression of vimentin but negative for cytokeratin. To address the fibroblasts phenotyping, immunofluorescence staining of vimentin and cytokeratin was performed. The cells showed high-expression of vimentin (Fig. 1A), but did not express cytokeratin (Fig. 1B).

### TGF-β1 activated the p38 signaling pathway in fibroblasts

**Figure 2:**
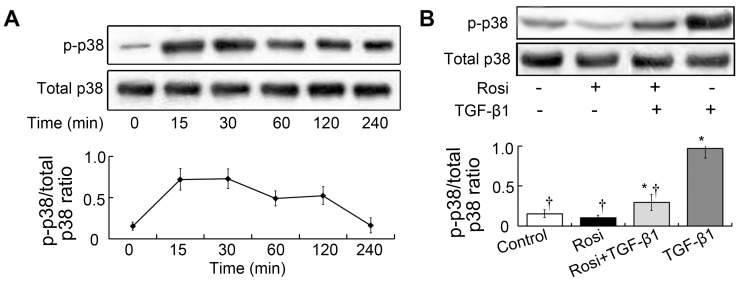
Rosiglitazone attenuated activation of the p38 signal pathway induced by TGF-β1. (**A**) TGF-β1 increased the expression of phosphorylated p38 in cultured fibroblasts. Cells were were stimulated with 5 ng/mL TGF-β1 under serum-free conditions. The phosphorylated p38 MAPK level was measured at various time points from 15 to 240 min. Phosphorylated p38 as measured by western blot increased after 15 min and peaked at 30 min. Total p38 expression was unchanged before and after stimulation with TGF-β1. This experiment was repeated 3 times with similar results. (**B**) Rosiglitazone exhibited no significant effect on the phosphorylation of p38 in unstimulated cells (*p*>0.05). However, the addition of rosiglitazone significantly suppressed p38 pathway activation in the cells stimulated with TGF-β1. Each bar (mean) is the average of repetitions of three repeated experiments, and the error bars shown the standard deviation. Asterisk indicates p<0.05 compared with control group; Dagger indicates p<0.05 compared with TGF-β1 group.

To address the involvement of p38 in activation of human Tenon fibroblasts, fibroblasts were stimulated with 5 ng/mL TGF-β1 under serum-free conditions. The phosphorylated p38 MAPK level was measured at various time points from 15 to 240 min. Phosphorylated p38 as measured by western blot increased after 15 min and peaked at 30 min (Fig. 2A). We also examined total p38 protein expression and found equal amounts of total p38 before and after stimulation with TGF-β1 (Fig. 2A). These results suggesting potential involvement of p38 in activation of fibroblasts in vitro.

### Rosiglitazone inhibited TGF-β1–induced activation of the p38 pathway

To assess the involvement of rosiglitazone in activation of p38, we studied the effects of rosiglitazone. Fibroblasts were treated with 10 µmol/L rosiglitazone for 2 h and then incubated with TGF-β1 under serum-free conditions. Phosphorylated p38 expression was measured 30 min after stimulation with TGF-β1. Rosiglitazone exhibited no significant effect on the phosphorylation of p38 in unstimulated cells (*p*>0.05; Fig. 2B). However, the addition of rosiglitazone significantly suppressed p38 pathway activation in the cells stimulated with TGF-β1 (*p*<0.05).

### Rosiglitazone and the p38 inhibitor SB20380 suppressed TGF-β1–induced fibroblast proliferation

**Figure 3:**
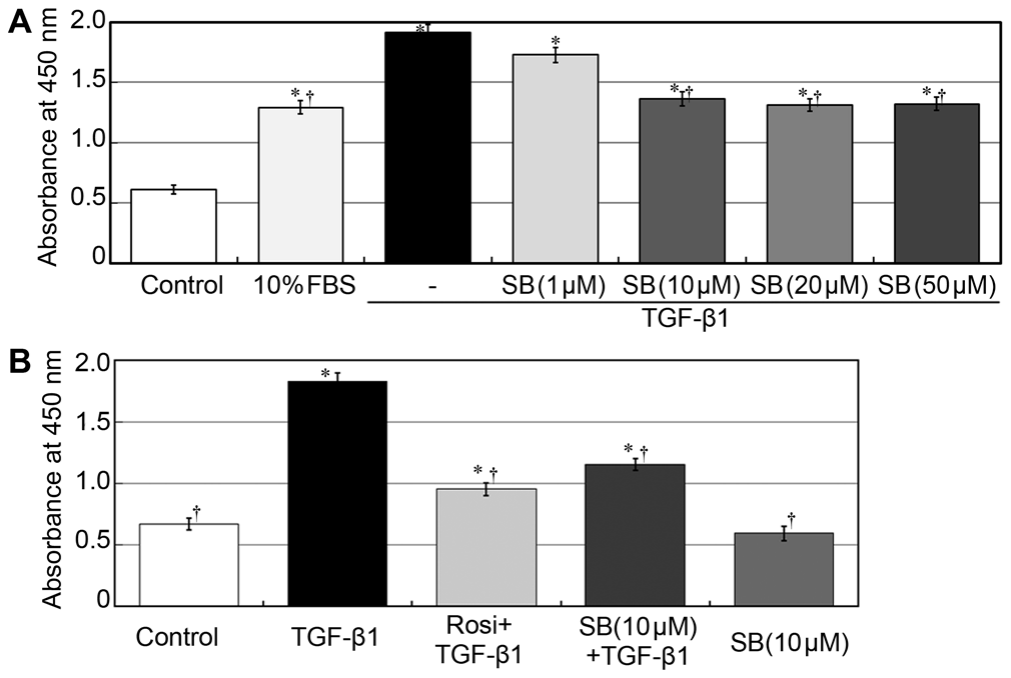
Rosiglitazone and SB203580 attenuated fibroblast proliferation induced by TGF-β1. (**A**) Cell Counting Kit-8 assays showed that treatment with TGF-β1 significantly increased the proliferation of fibroblasts after 24 h (*p*<0.05). While the TGF-β1-induced proliferation was inhibited by SB203580 in a dose-dependent manner (*p*<0.05). There is no significant differences among 10, 20 and 50 µmol/L (*p*>0.05). Cells were treated with TGF-β1 in the presence or absence of p38 specific inhibitor SB20380 at concentrations of 1, 10, 20 and 50 µmol/L. (**B**) SB203580 (10 µmol/L) alone exhibited no significant effect on fibroblast viability (*p*>0.05). The inhibitory effect of SB203580 on TGF-β1 stimulated cell proliferation was weaker than the effect of rosiglitazone (*p*<0.05). Each bar (mean) is the average of repetitions of three repeated experiments, and the error bars shown the standard deviation. Asterisk indicates p<0.05 compared with control group; Dagger indicates p<0.05 compared with TGF-β1 group.

To investigate whether proliferation of fibroblasts is p38-dependent, cells were treated with TGF-β1 in the presence or absence of p38 specific inhibitor SB20380 at concentrations of 1, 10, 20 and 50 µmol/L. Treatment with TGF-β1 significantly increased the proliferation of fibroblasts after 24 h (*p*<0.05; Fig. 3A). While the TGF-β1-induced proliferation was inhibited by SB203580 in a dose-dependent manner (*p*<0.05). There is no significant difference among 10, 20 and 50 µmol/L (*p*>0.05). Therefore, SB203580 at a concentration of 10 µmol/L was used in subsequent experiments.

SB203580 (10 µmol/L) alone exhibited no significant effect on fibroblast viability (*p*>0.05; Fig. 3B). However, pretreatment of rosiglitazone or SB203580 significantly suppressed TGF-β1 stimulated cell proliferation (*p*<0.05). The inhibitory effect of SB203580 on cell proliferation was weaker than the effect of rosiglitazone (*p*<0.05). Besides, both rosiglitazone and SB203580 treatment did not reduce proliferation to control level (*p*<0.05, respectively).

### Rosiglitazone and SB20380 attenuated TGF-β1–induced activation of fibroblast motility

**Figure 4:**
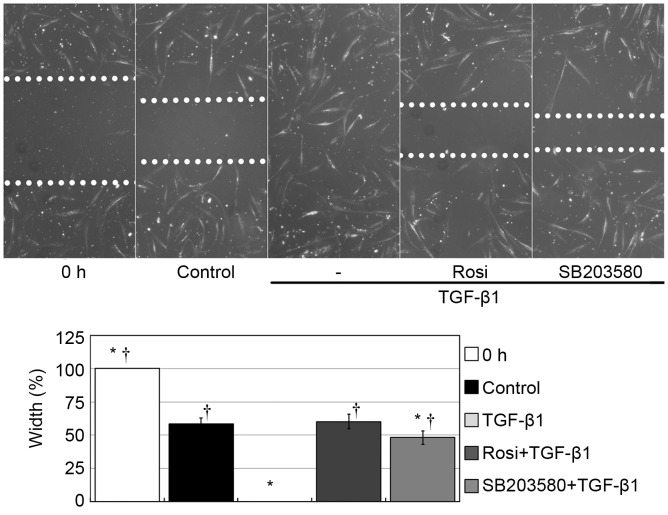
Rosiglitazone and SB203580 attenuated activation of fibroblast motility induced by TGF-β1. Stimulation with TGF-β1 significantly improved the fibroblast motility compared with control group (*p*<0.05). However, both rosiglitazone and SB203580 suppressed the enhancing effect of TGF-β1 on cell migration. There is no significant difference between rosiglitazone group and SB203580 group (*p*>0.05). Cells were seeded in six-well plates, and scratch wounds were performed. After washing away suspended particles, we preincubated the cells with rosiglitazone for 2 h or SB203580 for 1 h in serum-starved medium. Then, the cells were stimulated with 5 ng/mL TGF-β1 for another 24 h in serum-free medium in the presence of rosiglitazone or SB203580. Width of wound was measured at six different sites and averaged in each sample. The width in each group was expressed as a percentage of the original measurement before stimulation, and the original width was presented as 100%. Each bar (mean) is the average of repetitions of three repeated experiments, and the error bars shown the standard deviation. Asterisk indicates p<0.05 compared with control group; Dagger indicates p<0.05 compared with TGF-β1 group.

To address the possible involvement of the p38 signaling pathway in TGF-β1–induced cell migration, we studied the cell migratory ability after the application of SB203580. The edges of the exposed wounds were marked with dashed lines (Fig. 4). Twenty-four hours after stimulation, the wound width was 58% ±4% in the vehicle control, while the wound gap had disappeared in the TGF-β1 treatment group. The wound width was 60% ±5% in the cells pretreated with rosiglitazone and 48% ±5% in the cells pretreated with SB203580. The wound gaps significantly differed among the different treatment groups (*p*<0.05). Stimulation with TGF-β1 significantly improved the fibroblast motility compared with control group (*p*<0.05). However, both rosiglitazone and SB203580 suppressed the enhancing effect of TGF-β1 on cell migration. There is no significant difference between rosiglitazone group and SB203580 group (*p*>0.05), suggesting similiar inhibitory effect of rosiglitazone and SB203580 on fibroblast migration.

### TGF-β1–induced α-SMA, CTGF and Col I upregulation is p38 dependent

**Figure 5:**
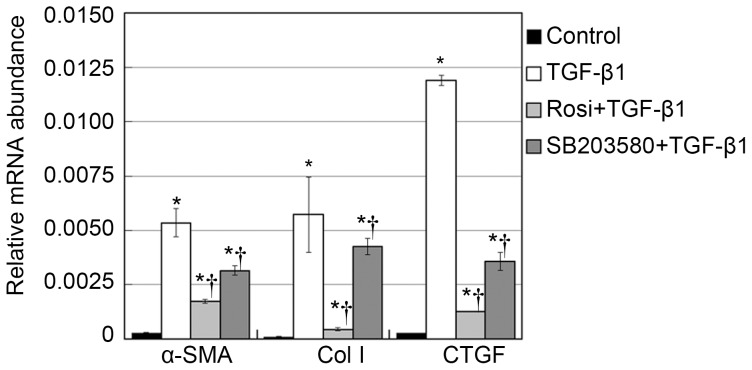
Rosiglitazone and SB203580 suppressed the mRNA expression of α-SMA, Col I, and CTGF. Fibroblasts were treated with rosiglitazone for 2 h or SB203580 for 1 h. Next, the cells were incubated with 5 ng/mL TGF-β1 in the presence of rosiglitazone or SB203580 for 12 h (for CTGF mRNA analysis) or 24 h (for α-SMA and Col I mRNA analysis). Fibroblasts treated with vehicle control expressed little α-SMA, Col I and CTGF, increased α-SMA, Col I and CTGF mRNA expression was observed in fibroblasts treated with TGF-β1 (*p*<0.05, respectively). Furthermore, additional SB203580 treatments significantly decreased the TGF-β1–induced increase in α-SMA mRNA expression (*p*<0.05). The expression of α-SMA in cells pretreated with rosiglitazone was lower than that in cells pretreated with SB203580 (*p*<0.05). Cells were treated with vehicle, rosiglitazone, SB203580, and TGF-β1. Next, the cells were incubated with 5 ng/mL TGF-β1 in the presence of rosiglitazone or SB203580. α-SMA mRNA abundance was analyzed qPCR, and the housekeeping gene GAPDH transcript was used for normalization. Each bar (mean) is the average of repetitions of three repeated experiments, and the error bars shown the standard deviation. Asterisk indicates p<0.05 compared with control group; Dagger indicates p<0.05 compared with TGF-β1 group.

In the wound healing process, extracellular matrix proteins such as collagen I and growth factors such as CTGF are abundantly synthesized after the transdifferentiation of fibroblasts to myofibroblasts. Myofibroblasts are characterized by high-expression of α-SMA [13]. Therefore, we studied the involvement of p38 in α-SMA, CTGF and Col I expression. α-SMA, CTGF and Col I mRNA levels were measured by qPCR. Although cells treated with vehicle control expressed little α-SMA, CTGF and Col I, increased α-SMA, CTGF and Col I mRNA expression was observed in fibroblasts treated with TGF-β1 (*p*<0.05, Fig. 5). Furthermore, additional SB203580 treatments significantly decreased the TGF-β1–induced increase in α-SMA, CTGF and Col I mRNA expression (*p*<0.05, Fig. 5).

**Figure 6:**
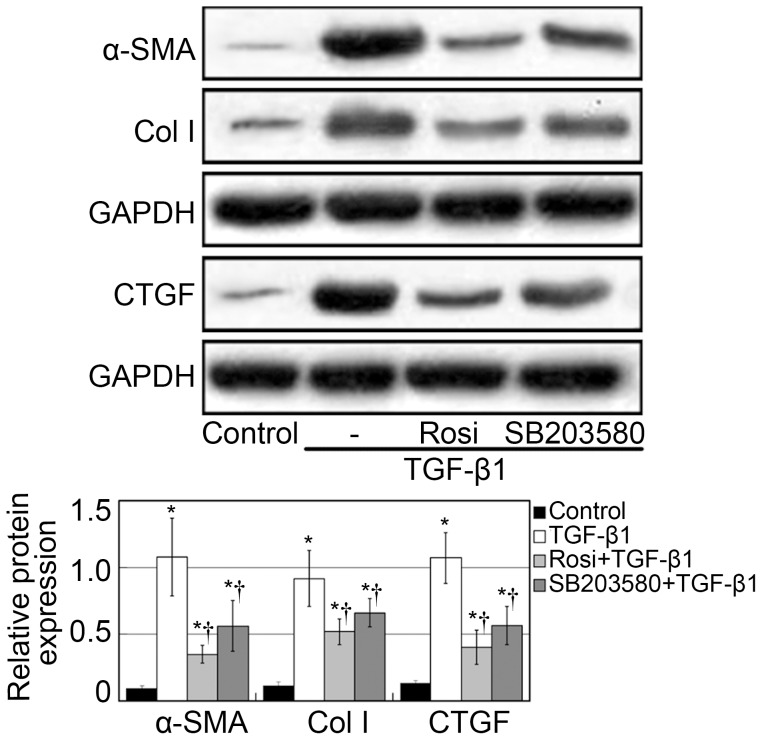
Rosiglitazone and SB203580 suppressed the protein expression of α-SMA, Col I, and CTGF. Fibroblasts were treated with rosiglitazone for 2 h or SB203580 for 1 h. Next, the cells were incubated with 5 ng/mL TGF-β1 in the presence of rosiglitazone or SB203580 for 24 h (for CTGF analysis) or 48 h (for α-SMA and Col I analysis). Fibroblasts treated with vehicle control expressed little α-SMA, CTGF and Col I, TGF-β1 stimulation upregulated protein expression of α-SMA, CTGF and Col I (*p*<0.05, respectively). The p38 MAPK inhibitor SB203580 clearly suppressed the TGF-β1–induced α-SMA, CTGF and Col I expression at the protein levels (*p*<0.05). The expression of α-SMA, CTGF and Col I in rosiglitazone group was lower than that in SB203580 group (*p*<0.05). Quantification of western blot products was expressed as the ratio between the optical density of target protein band and GAPDH band (target protein/GAPDH). Each bar (mean) is the average of repetitions of three repeated experiments, and the error bars shown the standard deviation. Asterisk indicates p<0.05 compared with control group; Dagger indicates p<0.05 compared with TGF-β1 group.

**Figure 7:**
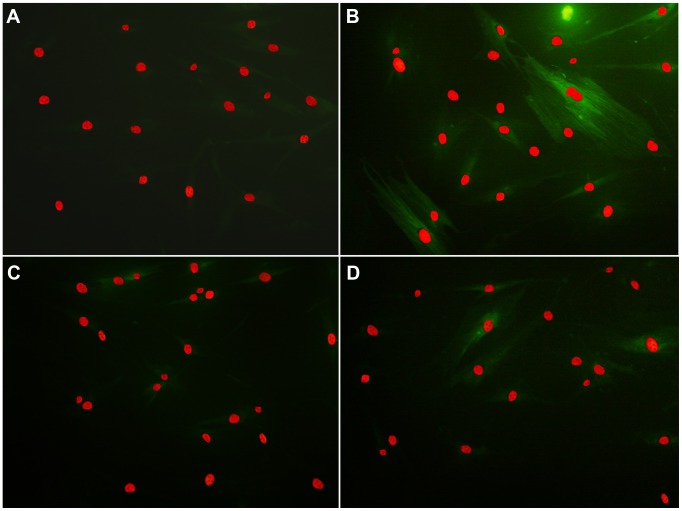
Recruitment of α-SMA in the cytoplasm of fibroblasts. Rosiglitazone and SB203580 attenuated the TGF-β1–induced upregulation of α-SMA: (**A**) vehicle control, (**B**) vehicle and TGF-β1, (**C**) rosiglitazone and TGF-β1, and (**D**) SB203580 and TGF-β1. Fibroblasts were preincubated with rosiglitazone or SB203580 in serum-starved medium. Then, the cells were incubated with 5 ng/mL TGF-β1 in the presence of rosiglitazone or SB203580 for another 48 h. Cells analyzed by immunofluorescence with α-SMA (green) as a representative fibrosis marker and propidium iodide to stain nuclear (red).

To further quantify the effects of p38 on α-SMA, CTGF and Col I expression, western blot assays were performed. The p38 MAPK inhibitor SB203580 clearly suppressed the TGF-β1–induced upregulation of α-SMA, CTGF and Col I protein expression (*p*<0.05; Fig. 6). Therefore, p38 seems to have contributed to TGF-β1–induced α-SMA, CTGF and Col I expression in human Tenon fibroblasts. Furthermore, the expression of α-SMA, CTGF and Col I in rosiglitazone group was lower than that in cells pretreated with SB203580 (*p*<0.05). Similar results were found on immunofluorescence microscopic evaluation of α-SMA expression (Fig. 7). These findings further support the critical involvement of p38 in antifibrotic effect of rosiglitazone.

## Discussion

Filtration surgery remains one of the most important treatments for glaucoma. However, postoperative scarring of the filtration passage is often associated with failure of the surgery [14]. The use of anti-metabolites such as 5-fluoruoracil and mitomycin C has improved the success rate of this surgery. However, these drugs are often associated with serious postoperative complications, such as bleb leakage, hypotony maculopathy, and endophthalmitis [15], [16]. Therefore, new specific agents with fewer adverse effects are required to overcome this therapeutic shortcoming.

It has been reported that p38 MAPK is involved in scar formation [17]. TGF-β1 stimulation increases the expression of phosphorylated p38 MAPK in periodontal ligament fibroblasts [18]. Phosphorylation of p38 MAPK is also critical for the induction of Col I production by TGF-β1 in dermal fibroblasts [19]. Hence, targeting p38 may be an attractive therapeutic approach for reducing specific aspects of the fibrotic response at the level of the fibroblast. In this study, we found that the p38 MAPK signaling pathway is activated in human Tenon fibroblasts stimulated with TGF-β1. The time to peak p38 phosphorylation was 30 min in our study, which is similar to the finding in human corneal fibroblasts [20]. These results suggest that the same mechanism is involved in scarring in Tenon’s capsule and the cornea.

The p38 inhibitor SB203580 did not influence fibroblast viability and motility for up to 24 h. However, 10 µmol/L SB203580 significantly suppressed the enhancing effect of TGF-β1 on fibroblast proliferation and migration. We also found that α-SMA expression in fibroblasts was significantly suppressed by SB203580. Since myofibroblasts are characterized by high-expression of α-SMA, it is reasonable to propose that the p38 signaling pathway is involved in the activation of human Tenon fibroblasts induced by TGF-β1.

Excess deposition of extracellular matrix is the key characteristic of fibrosis in various tissues. Collagens, especially Col I, are the major components of the extracellular matrix. CTGF is an important downstream signaling molecule of TGF-β1. Our present findings showed that SB203580 abrogated the TGF-β1–induced overexpression of Col I and CTGF. Our observation is in accordance with a study of conjunctival fibroblasts in mice, in which the introduction of a dominant-negative p38 MAPK gene inhibited TGF-β1–induced CTGF and Col I expression [6]. These results indicate that TGF-β1 activates wound healing, but blocking of p38 may prevent deposition of the extracellular matrix during wound healing.

A wide variety of p38 inhibitors are currently being researched and developed [21], [22], with progress in affinity and bioactivity [23]. However, none of these agents can be used in the clinical setting. Rosiglitazone is known for its ability to improve insulin sensitivity and has been used extensively in patients with non-insulin-dependent diabetes mellitus. However, rosiglitazone has been proven to have an anti-fibrotic effect in multiple organs [24]–[26], and we previously demonstrated that rosiglitazone exerts its anti-fibrotic effect through suppression of Smad2/3 [12]. In this study, we investigated the effect of rosiglitazone on the p38 signaling pathway. Our results demonstrated that the increase in the level of phosphorylated p38 after TGF-β1 treatment was substantially abrogated by rosiglitazone. Moreover, the anti-fibrotic mechanism of rosiglitazone involves interference with the TGF-β/p38 signaling pathway. However, rosiglitazone had a stronger effect on activation of fibroblasts than did the p38 inhibitor SB203580, which suggests that rosiglitazone exerts its anti-fibrotic effect through multiple signaling pathways, one of which is the p38 pathway. This is consistent with our previous study that inhibitory effect of rosiglitazone on smad2/3 decreased activation of fibroblasts. But rosiglitazone and SB203580 suppressed TGF-β1–induced fibroblast migration at a similar level, suggesting that the p38 plays a stronger role in cell motility than transdifferentiation.

Neither rosiglitazone nor p38 inhibitor SB203580 completely abrogated the transdifferentiation of Tenon fibroblasts to myofibroblasts, suggesting a complex regulatory link between TGF-β and fibrosis. Blocking of p38 did not decreased α-SMA, CTGF and Col I to a control level, which indicates that TGF-β1–induced fibrosis is only partially p38 dependent. Previous studies have indicated the reciprocal regulation (positive or negative) of activated Smads and p38 MAPK. We have previously shown that smad2/3 mediated TGF-β1–induced fibrosis. In this study, blocking of the p38 causes an anti-fibrotic effect. These data indicate that TGF-β–induced fibrosis requires activation of both Smad2/3 and p38 MAPK for optimal transdifferentiation.

MAPK represent a major signaling pathway for TGF-β independent of Smads. We showed that the Smad and the p38 MAPK pathways integrate in fibroblasts transdifferentiation. In some cases, activation of p38 by TGF-β is slow, indicating that it may be delayed or indirect effect, perhaps resulting from Smad-dependent transcriptional reponse [5]. However, the activation in our study is very rapid, implying that it might be through a direct post-translational modification. Collectively our data suggest that activation of both p38 and Smad2/3 MAPK is required to induce fibrosis by TGF-β1.

Despite these findings, the underlying mechanism and biological consequence of smad2/3 and p38 activation are not clear. The possible interaction among smad2/3, p38 and PPAR-γ require further investigation.

We have shown the fibrosis suppressor properties of rosiglitazone are likely mediated in part via suppression of p38. Although rosiglitazone does not appear to totally block activation of fibroblasts, its ability to attenuate fibrosis may be relevant in the scarring of the filtration passage. We conclude that rosiglitazone is a potential therapeutic agent in glaucoma filtration surgery.
